# Gut Microbiota and Metabolic Specificity in Ulcerative Colitis and Crohn's Disease

**DOI:** 10.3389/fmed.2020.606298

**Published:** 2020-11-27

**Authors:** Jagadesan Sankarasubramanian, Rizwan Ahmad, Nagavardhini Avuthu, Amar B. Singh, Chittibabu Guda

**Affiliations:** ^1^Department of Genetics Cell Biology and Anatomy, University of Nebraska Medical Center, Omaha, NE, United States; ^2^Department of Biochemistry and Molecular Biology, University of Nebraska Medical Center, Omaha, NE, United States; ^3^VA Nebraska-Western Iowa Health Care System, Omaha, NE, United States

**Keywords:** gut microbiome, metabolism, ulcerative colitis, Crohn's disease, prognosis

## Abstract

**Background:** Inflammatory bowel disease (IBD) represents multifactorial chronic inflammatory conditions in the gastrointestinal tract and includes Crohn's disease (CD) and ulcerative colitis (UC). Despite similarities in pathobiology and disease symptoms, UC and CD represent distinct diseases and exhibit diverse therapeutic responses. While studies have now confirmed that IBD is associated with dramatic changes in the gut microbiota, specific changes in the gut microbiome and associated metabolic effects on the host due to CD and UC are less well-understood.

**Methods:** To address this knowledge gap, we performed an extensive unbiased meta-analysis of the gut microbiome data from five different IBD patient cohorts from five different countries using QIIME2, DIAMOND, and STAMP bioinformatics platforms. *In-silico* profiling of the metabolic pathways and community metabolic modeling were carried out to identify disease-specific association of the metabolic fluxes and signaling pathways.

**Results:** Our results demonstrated a highly conserved gut microbiota community between healthy individuals and IBD patients at higher phylogenetic levels. However, at or below the order level in the taxonomic rank, we found significant disease-specific alterations. Similarly, we identified differential enrichment of the metabolic pathways in CD and UC, which included enriched pathways related to amino acid and glycan biosynthesis and metabolism, in addition to other metabolic pathways.

**Conclusions:** In conclusion, this study highlights the prospects of harnessing the gut microbiota to improve understanding of the etiology of CD and UC and to develop novel prognostic, and therapeutic approaches.

## Introduction

Inflammatory Bowel Diseases (IBDs) consist of a series of autoimmune chronic inflammatory conditions of the gut and include Crohn's Disease (CD) and Ulcerative Colitis (UC) (1). The hallmark of both IBDs is inflammation. Also, CD and UC share disease symptoms, including diarrhea, abdominal pain, and weight loss. However, despite the symptomological similarities, CD and UC have quite distinct pathobiology regarding the spatial distribution and penetrance of inflammation along the intestine and therapeutic responses (2). In the United States, CD and UC affect ~1 person in every 200 people (3) and a 5–10 and 2–10 fold increase has been noted in the prevalence of CD and UC, respectively, in developed countries over the past decade (4).

While, the etiology of IBD is not well-understood, environmental factors and the host genetics play important roles in regulating the disease's pathology and prognosis (1, 5). Here, one of the most recognized theories is that abnormal immunological responses to the gut microbiota play a central role in IBD susceptibility and progression. In this regard, recent studies have demonstrated that the gut microbiota acts as a metabolic organ and contributes to human health by active participation in various physiological functions of the host (6). Accordingly, composition of the gut microbial communities is critically different between healthy individuals and IBD patients (7). Such compositional changes of the gut microbiota, commonly referred to as “gut dysbiosis,” are now being comprehended for developing promising strategies for prognosis and treatment of the disease (8). However, it remains unclear whether gut dysbiosis associated with the CD and UC is disease-specific, as it may help develop accurate disease predictive and management models. Moreover, an improved understanding of such differences and associated metabolic changes may help in devising novel therapeutic intervention strategies.

The current study was aimed at addressing the above described knowledge gaps. We examined fecal metagenomics sequencing data derived from CD and UC patients from five developed countries with known prevalence of IBD. The fecal metagenomics data and associated disease metadata were analyzed to identify microbial associations with CD, UC and healthy controls. Outcomes from these analyses were then subjected to “*in silico*” community modeling and metabolic pathway construction. Overall, despite the known diversity of the gut microbial communities, we found consistent differences between the gut microbiota of CD and UC patients. The gut microbial metabolic modeling further suggested disease specificity in the microbial metabolic fluxes/pathways for CD vs. UC. We believe these findings aid in the current understanding of microbial dysbiosis in CD and UC patients and toward development of effective diagnostic and therapeutic strategies.

## Materials and Methods

### Data Collection

Fecal metagenomics sequencing data from IBD patients (CD and UC) and corresponding healthy controls (HC) were retrieved from the National Center for Biotechnology Information (NCBI). We used five different datasets belonging to the IBD patients from developed countries including USA, Canada, and three European countries (UK, Spain, and Netherlands). Among these, four datasets were generated using 16S rRNA gene amplicon sequencing while the fifth dataset was generated using the whole metagenome sequencing [NCBI SRA accession: SRP129027] (9). The NCBI SRA accession numbers for the four 16S rRNA datasets are: SRP183770 (10), SRP128892 (11), SRP115494 (12), and ERP008725 (13). The criterion in the selection of these datasets was that each dataset must contain data from at least 20 subjects each from the CD, UC and healthy cohorts. Details of samples used for the analysis from these five datasets are provided in Supplementary Figure 1.

### Metagenomic Data Analysis

Raw sequencing reads (fastq files) from publicly available datasets were analyzed using QIIME2 (Quantitative Insights Into Microbial Ecology version 2) software, a next-generation microbiome bioinformatics platform to determine the taxonomic diversity profiles of the microbiota in healthy and IBD samples (14). The QIIME2 plugin, DADA2 algorithm was used for quality-score based filtering of the input sequences and construction of feature table, which also contains the count of each unique sequence of each sample. To assign the taxonomy of the Feature Data (unique sequences), the pre-trained Naive Bayes and q2-feature classifiers were used. The sequences were clustered into Operational Taxonomic Units (OTUs) using a closed-reference OTU picking workflow against the Greengenes (15) 13_8 reference set from V4 region, based on an average percent identity of 99%. To avoid the problem of spurious OTUs, the singletons and doubletons were removed, and the ultimate counts/sample were generated. The whole metagenome dataset SRP129027 was aligned using DIAMOND (16) against the full NCBI NR database, which uses the “seed and extend” method to find all matches between a query sample and the reference database. The aligned sample data was saved in a compressed format called DAA (DIAMOND alignment archive). DAA files were then imported into the MEGAN6 (17) for functional classification using InterPro2GO, eggNOG, KEGG, and SEED classification schemes.

### Comparison of the Five Different Datasets

The alpha diversity (Shannon diversity) and beta diversity (Bray-Curtis distance) of all the IBD datasets were calculated and plotted using VEGAN R package (18) based on relative frequency of taxonomic profiles. The diversity of statistically significant species between HC, UC, and CD was assessed using Wilcoxon rank-sum tests and corrected for multiple testing hypothesis (Benjamini-Hochberg method) with the *p*-value <0.05 considered as statistically significant. The differential microbial features for HC vs. IBD, HC vs. CD, HC vs. UC and CD vs. UC in all the five datasets were identified using Statistical Analysis of Metagenomic Profiles (STAMP; v2.1.3) (19) software. The differential taxa (at order level) identified from all the datasets were plotted using UpSetR (20) to show the microbial taxa shared among the datasets. For metabolic modeling of HC, CD, and UC microbial communities, we selected the differential microbial species that were present in at least three of the five datasets to avoid the biasness based on the dataset.

### Pan-Genome Analysis and Metabolic Model Construction

A total of 12 significant microbial species were identified in our meta-analysis as differential taxa among the HC, CD, and UC comparisons. To identify the metabolic fluxes of these differentiating taxa in HC, CD, and UC gut, we performed *in silico* metabolic modeling. For this, we retrieved the complete genome or draft genome sequences of 12 differentiating taxa from NCBI. For the draft genome, the strain that has the lower number of contigs with the highest fold coverage in a particular species was taken and used for the further analysis. Thereafter, we predicted the similarity between the bacterial genomes using Gegenees (21), which uses a fragmented alignment approach to facilitate the comparative analysis of microbial genomes. As proposed by Tettelin et al., a pan-genome can be defined as being the entire gene content of all strains in the study group (22). Thus, the Pan-genome consisted of the core genome, accessory or dispensable genome as well as unique or novel genome. Genes present in all microbial strains were considered as the core genome, and those missing in at least one strain of a microbial species were called the accessory genome, while genes present only in a single strain were considered unique. *KBase* (23) is a collaborative, open environment platform for studying the systems biology of plants, microbes, and their communities. It also has several analysis tools and data for systems biology. The *Compute Pan-genome* (v.0.07) and *Compare Genomes from pan-genome* (v.0.07) tools from KBase were used for the pan-genome construction. For disease-specific microbes, metabolic models were built using the *Build Metabolic Model* (v.1.7.6) tool from the KBase. In the metabolic modeling, bacterial growth rates were determined using *in silico* methods; we used the biological media as complete media or default media in *KBase* to construct the gap-fill model. The constructed 12 metabolic models were then compared using the *Compare Model* (v.1.7.6) app from *KBase*, which helps identify pan-genes, pan-reactions, pan-metabolites involved in disease-related microbes.

### Integrating the Metabolic Model Into the Community Model

Metabolic models were constructed for all three groups (CD, UC and HC), where each group contained four group-specific microbes. We then used the KBase tool *Merge Metabolic Model into Community Model v.1.7.6* to construct three community models, where similar reactions among the four microbes within each group were merged by a mixed-bag model. After building three community models, we performed the flux balance analysis in KBase using *Run Flux Balance Analysis v.1.7.6*, with the default media and Biomass reaction to predict metabolic fluxes in a metabolic model. Then, we identified the reactions with flux values that are involved in pathways.

### Statistical Analyses

OTU tables were used for downstream analysis to identify the functional and taxonomic profiles. Data were further analyzed using the following statistical methods: STAMP; v2.1.3 (19) software package was used to estimate the diversity of microbial communities between: (i) HC and IBD samples; (ii) CD and UC samples; and (iii) HC, CD and UC samples. For comparison between the two specific groups, for example: HC vs. IBD and CD vs. UC, Welch's *t*-test was applied. To predict the effect size and confidence intervals, the differences in mean proportion effect size measure along with Welch's confidence intervals were used. ANOVA was done for statistical comparison of the data from multiple groups, i.e., CD vs. HC vs. UC. Statistically significant features were examined using *post-hoc* tests (e.g., Tukey–Kramer) to determine how CD vs. HC vs. UC profiles differ from each other. Eta-squared effect size measure was used to predict the effect size (<0.80) and confidence intervals. To determine the false discovery rate (FDR), the multiple test correction method, Benjamini-Hochberg was used in all the comparisons. A statistical difference of at least *P* < 0.05 was used to select the significant features within a group of profiles.

### Datasets Used for Validation

For validation purposes two different whole metagenomic datasets consisting of CD, UC, and HC samples that were generated from subjects in USA were used. These datasets were retrieved from NCBI SRA SRP108708 (24) and SRP115812 (25), which consists of 157 and 300 samples, respectively. These datasets were processed using DIAMOND, MEGAN and STAMP packages using the same parameters as described above.

## Results

**Figure 1:**
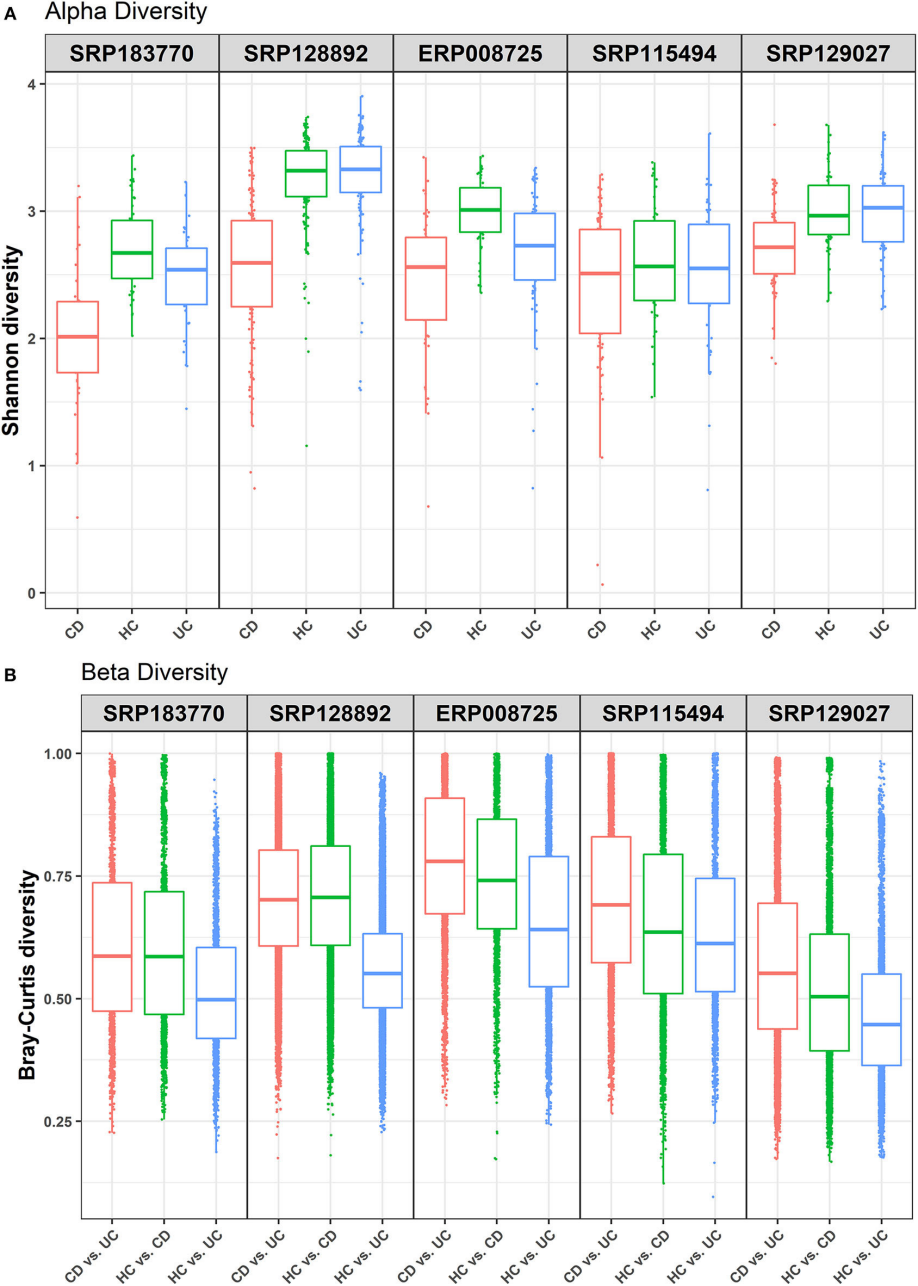
Alpha and beta diversity comparisons among HC, CD and UC cohorts. Analyses were performed on species-level taxa. **(A)** Boxplot showing Shannon diversity of each group. Each dot represents a sample and the lines in the boxes correspond to the median of samples; **(B)** Bray–Curtis distances between the comparison pair. Dots represent the distance between the samples in each comparison group and the lines in the boxes correspond to the median.

This study was undertaken in view of the established fact that gut dysbiosis promotes susceptibility to IBD and disease severity. However, significance of this causal association for disease specificity for the CD and UC and molecular modalities of the host-microbe interaction remain poorly understood. Overall, we attempted to address the following critical questions: (i) how conserved are the gut microbial communities among IBD patients; (ii) whether gut dysbiosis precipitates in a disease-specific manner in UC and CD; and (iii) whether gut dysbiosis has disease-specific effects on the host metabolism. We focused on the meta-analysis of published raw sequenced data on gut microbiome from matched cohorts of healthy and IBD-patients from developed countries including the USA, Canada, Spain, UK, and Netherlands (Supplementary Figure 1). All these datasets were retrieved from NCBI to our local server for the meta-analysis. Each dataset was individually analyzed and compared in four pair-wise combinations (i.e., IBD vs. HC, CD vs. UC, CD vs. UC vs. HC), to predict the specific microbes associated with healthy control and/or IBD, based on the statistical FDR *p*-value (<0.05). To reduce false positives, we followed stringent criteria and focused only on those microbial species that were conserved in at least three of the five datasets analyzed. The alpha diversity, as measured by the Shannon diversity index, was determined using the number and types of observed OTUs within each dataset (Figure 1A). The Shannon index increases as both the richness and evenness of the community increases. In most cases the HC group showed higher Shannon diversity over both the CD and UC groups, and UC recorded higher diversity over CD. In contrast, the diversity index was relatively uniform across all three groups in the SRP115494 dataset. We also calculated the beta diversity between the groups using Bray-Curtis distance measure for HC vs. CD, HC vs. UC and CD vs. UC groups to understand the level of species overlap between the groups. Beta diversity was smaller when there was more overlap of species between groups, and vice-versa. In all five datasets, beta diversity between HC vs. UC was lower compared to HC and CD, indicating that there are more overlapping species in UC with HC than in CD with HC (Figure 1B). On the other hand, CD vs. UC had consistently showed higher beta diversity indicating very low overlap of species between these two groups.

### Gut Microbial Composition in IBD Significantly Differ From That of Controls

**Figure 2:**
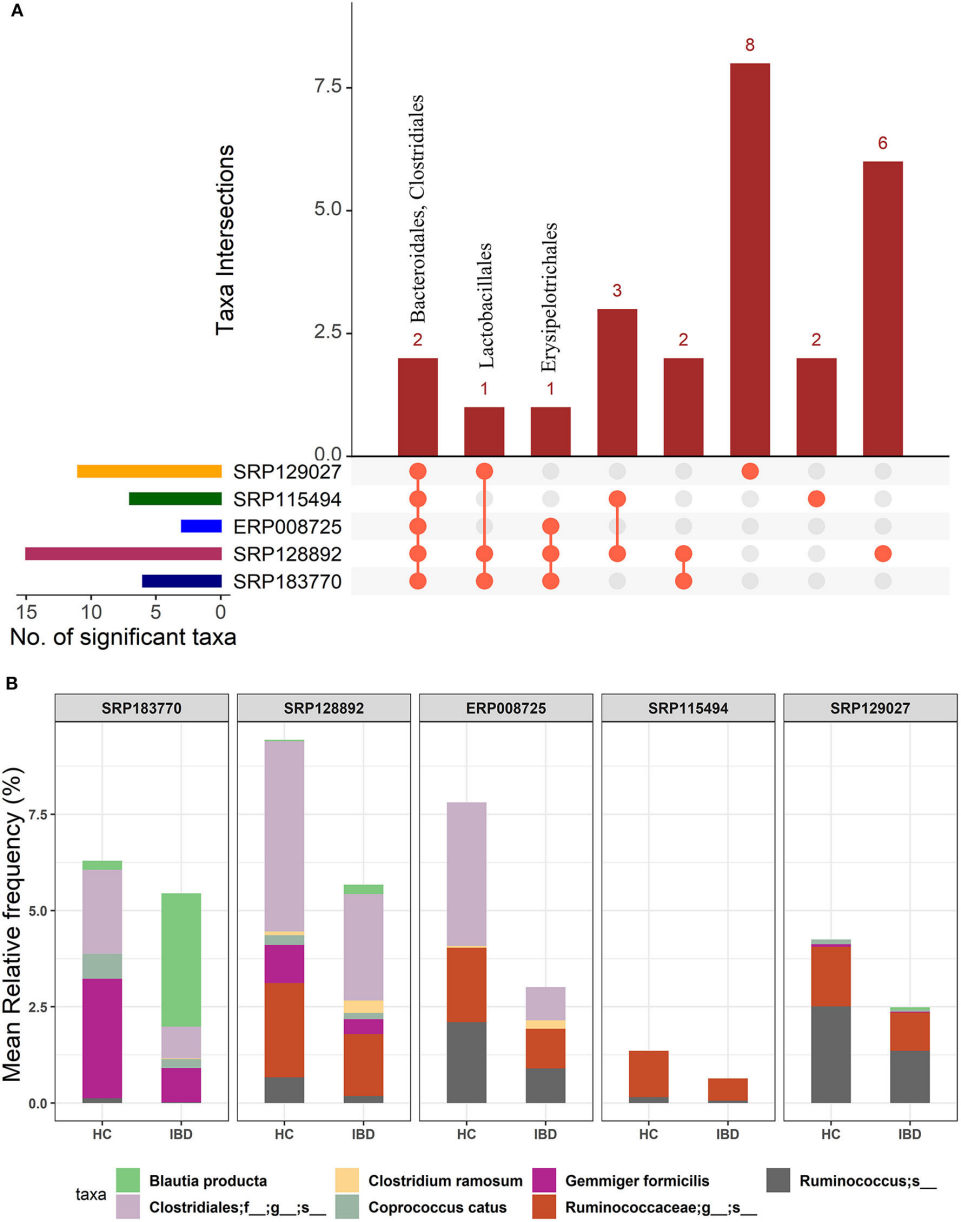
Comparison of microbial communities between IBD and HC cohorts across five different datasets. **(A)** An upset plot showing taxonomic intersections across the five datasets at the Order-level. Each bar represents the number of orders in that category and the orange dot below the bar indicates their conservation across the datasets. For instance, members of Bacteriodales and Clostridiales are conserved in all five datasets; **(B)** Stacked bar plots show the relative mean frequencies of significant species-level communities in IBD or HC that are present in at least in three out of five datasets. Corresponding values are provided in the 146 OTUs sheet in Supplementary Datasheet 3, where the columns contain data for five different datasets.

We first performed an unbiased analysis of the five datasets by comparing the gut microbiota of healthy controls against all IBD patients (including all CD and UC patients). We analyzed the order-level OTUs and identified 25 orders across five datasets that were significantly different (FDR corrected *p*-value <0.05) between the healthy controls and IBD patients (Supplementary Datasheet 2). Out of these, members of two orders, Bacteroidales and Clostridiales were conserved in all five datasets while members of Lactobacillales and Erysipelotrichales were conserved in at least three datasets (Figure 2A). Of note, we classified all the significant OTUs from the kingdom to the species level in these datasets (Supplementary Table 1), but only order-level differences were used to compare between the IBD vs. the HC groups (Figure 2A). Further analysis revealed more significant differences between HC and IBD at the species-level with number of significant species ranging from 11 to 63 across all five datasets analyzed (Supplementary Table 2 and Supplementary Datasheet 3). A combined total of 146 unique species were identified to be significantly different between the HC and IBD group; however, only seven of them were conserved in at least three of the five datasets. The mean relative frequencies of these seven species were then compared between the HC and IBD groups (Figure 2B). Microbial species such as *Gemmiger formicilis* (*p*-value = 1.51^e−8^) and those from the order *Clostridiales* were highly enriched in the HC group compared to the IBD groups. Similarly, microbial species from family *Ruminococcaceae*, in specific, from genus *Ruminococcus* showed significantly high abundance in HC compared to the IBD (*p*-value = 8.66^e−4^). In contrast, *Blautia producta* (*p*-value = 6.75^e−4^) and *Clostridium ramosum* (*p*-value = 8.86^e−5^) were highly enriched in IBD compared to the HC group (Supplementary Datasheet 3). Overall, above analyses confirmed the existence of major differences in the diversity and abundance of the gut microbial communities between healthy individuals and IBD patients.

### Microbial Species Specificity for CD and UC Patients Compared to the Healthy Individuals

**Figure 3:**
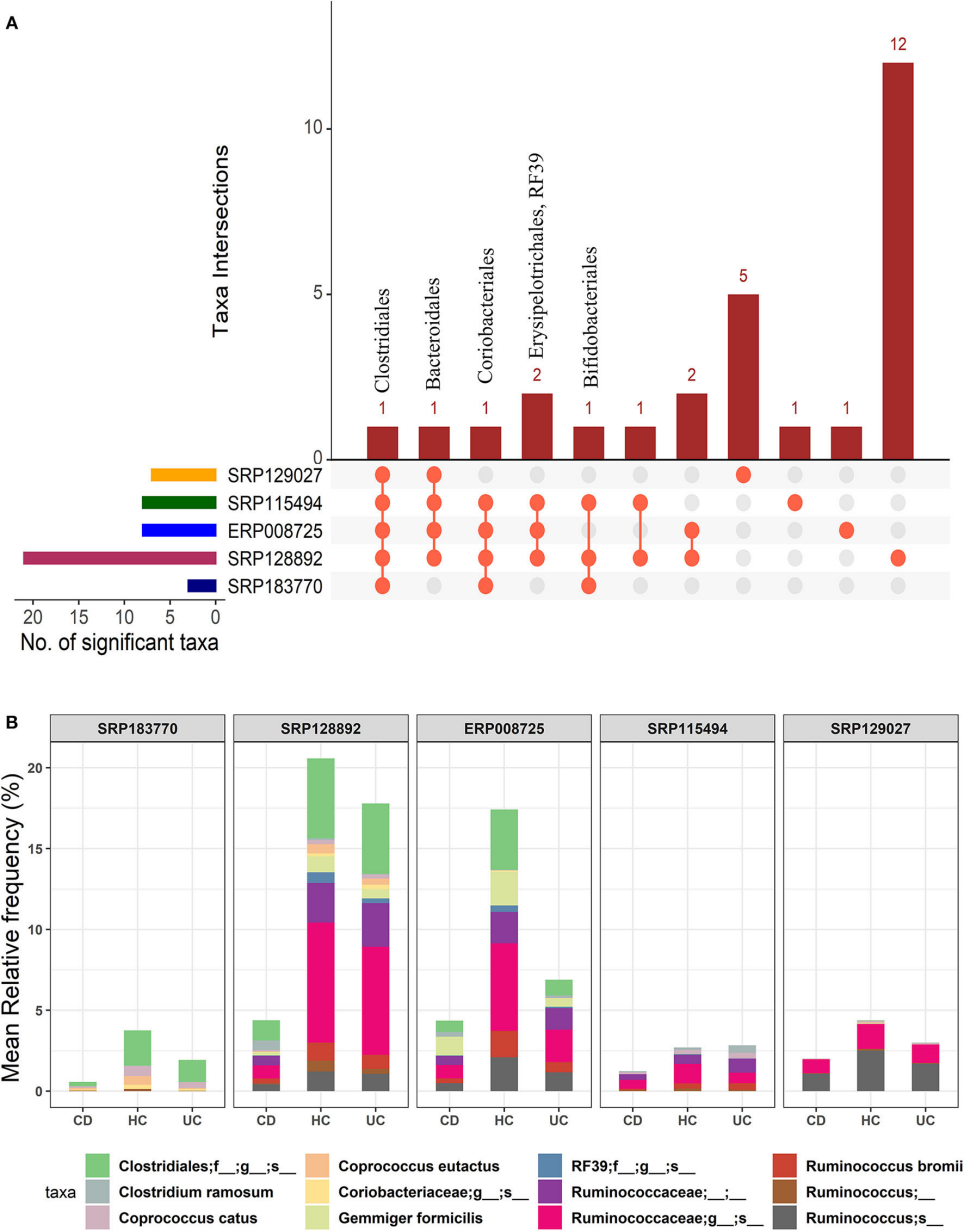
Comparison of microbial communities among CD, UC, and HC cohorts across five datasets. **(A)** An upset plot showing taxonomic intersections across the five datasets at the Order-level. Each bar represents the number of orders in that category and the orange dot below the bar indicates their conservation across the datasets. For instance, members of Clostridiales are conserved in all five datasets; **(B)** Stacked bar plots show the relative mean frequencies of significant species-level communities in CD, HC or UC that are present in at least in three out of five datasets. Corresponding values are provided in the 168 OTUs sheet in Supplementary Datasheet 4, where the columns contain data for five different datasets.

In the light of above findings, we wondered if disease-specificity of the gut microbiota in UC and CD patients will persist even when compared with the gut microbial composition in the HC group. To this end, IBD patients from all five datasets were divided into the CD or UC cohorts using the corresponding tags in the metadata. A multi-group analysis was done while keeping the parameters for inclusion/exclusion of specific microbes the same as above. In this comparison, we identified 28 OTUs at the order-level taxa (Supplementary Datasheet 2). However, members of only one order, Clostridiales, were found to be conserved in all five datasets. The members of the Bacteroidales and Coriobacteriales were found to be conserved in four datasets while those belonging to the Bifidobacteriales, Erysipelotrichales and RF39 were identified in at least three datasets (Figure 3A). Similarly, below the order level we found higher divergence. These OTU distributions from the kingdom to species level are provided in the Supplementary Table 1. Overall, this comparison predicted 10 to 109 significant OTUs across the five datasets at the species-level (Supplementary Table 2) with a total of 168 unique OTUs (Supplementary Datasheet 4). Out of these, 12 OTUs were identified as conserved (present in at least three datasets) (Figure 3B). In particular, the species *G. formicilis* and *Coprococcus catus* were highly enriched in HC when compared to the IBD patients (Figure 3B and Supplementary Datasheet 4). The species *C. ramosum* (*p*-value = 2.64^*e*−19^) however showed a significant enrichment in the CD patients (Supplementary Datasheet 4). The *Caprococcus eutatus, Ruminococus bromii* and *G. formicilis* were all highly enriched in CD patients compared with the HC samples (Supplementary Datasheet 4). Notably, these organisms play a significant role in distinguishing healthy patients from IBD patients.

Overall, we identified 12 unique microbial species in our multi-group analysis, which included four differentiating species for each: the CD, UC, and HC cohorts, as listed in the Supplementary Table 3. The species that showed significant association with the HC included *C. catus, C. eutatus, R. bromii*, and *G. formicilis*. The CD-specific organisms included the *C. ramosum, Ruminococcus lactaris*, and *Clostridium clostridioforme* and *Clostridium bolteae, two* species that belonged to the genus *Clostridium* and family Lachnospiraceae. Similarly, the four differentiating microbial species that showed significant association with UC included the *Ruminococcus albus, Ruminococcus callidus, Faecalibacterium prausnitzii*, and *Clostridium celatum*.

### Disease-Specific Microbial Association in CD vs. UC

**Figure 4:**
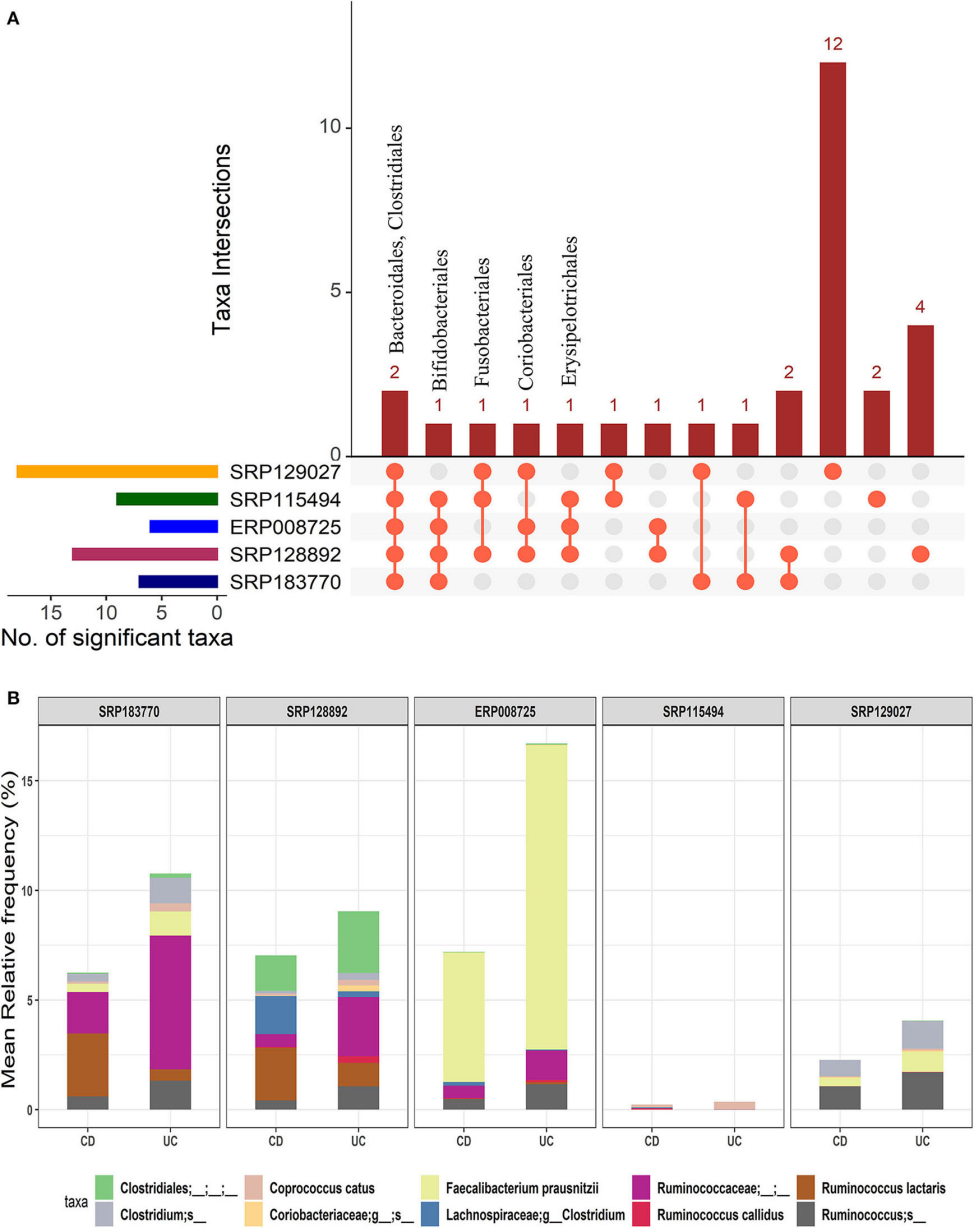
Comparison of microbial communities between CD and UC cohorts across five datasets. **(A)** An upset plot showing taxonomic intersections across the five datasets at the Order-level. Each bar represents the number of orders in that category and the orange dot below the bar indicates their conservation across the datasets. For instance, members of Bacteroidales and Clostridiales are conserved in all five datasets; **(B)** Stacked bar plots show the relative mean frequencies of significant species-level communities in CD or UC that are present in at least in three out of five datasets. Corresponding values are provided in the 195 OTUs sheet in Supplementary Datasheet 5, where the columns contain data for five different datasets.

We further investigated how microbial communities differ between CD and UC patients. At the order-level, a total of 30 OTUs were identified as significantly different in the CD cohort vs. the UC cohort (corrected *p*-value ≤0.05) (Supplementary Datasheet 2). Similar to the IBD vs. HC comparison, both *Bacteroidales* and *Clostridiales* were conserved in all five datasets. Likewise, Bifidobacteriales were conserved in four datasets while Coriobacteriales, Erysipelotrichales, and Fusobacteriales were present in at least three datasets (Figure 4A). However, this analysis showed higher levels of divergence from kingdom to the species level comparison (Supplementary Table 1). Further analysis revealed a cluster of 21-88 OTUs to be significantly different in CD vs. UC at the species level (Supplementary Table 2 and Supplementary Datasheet 5). From the five datasets combined, a total of 195 OTUs were predicted to be significantly different between the CD and UC cohorts. Among these, ten OTUs were identified as conserved, based on the criteria that an OTU must be present in at least three of the five datasets examined (Figure 4B and Supplementary Table 2). Importantly, we found that the members of genus *Clostridium* belonging to two different families, Lachnospiraceae and Clostridiaceae, were rather specific for CD or UC, respectively. The genome sizes of the members of the genus *Clostridium* also varied, depending on the family they belong to (Table 1). Similarly, members of the genus *Ruminococcus* also belonged to multiple families; their disease-specific association was distinguishable by their family, Lachnospiraceae and Ruminococcaceae in CD and UC, respectively (Supplementary Datasheet 5). At the species level, *R. lactaris* (from family, 2.8% higher relative frequency (*p*-value = 0.016) in CD compared to UC (Supplementary Datasheet 5). In addition, *C. catus, R. callidus*, and *F. prausnitzii* were also able to differentiate the UC patients from CD patients at a statistically significant threshold level (Supplementary Datasheet 5). Similar trends were seen for the *Lachnospiracae* and *Ruminococcaceae* families as they were decreased in the CD patients in comparison with the UC patients, while *Ruminococcus gnavus* was increased vice versa (Supplementary Datasheet 5). Overall, these studies helped designate typical changes in the composition of gut microbial composition in UC vs. CD patients.

**Table 1:** Metabolic characterization of disease-specific species involved in CD, HC, and UC.

**Diseases**	**Species**	**Accession no.**	**Strain type**	**Size**	**Total genes**	**Reactions**	**Metabolites**	**Genes in Model**	**FBA^#^**	Reactions^*^	**Metabolites**	**FBA^#^**
CD	*Clostridium bolteae* ATCC BAA-613	ABCC00000000	Gram-positive	~6.6	6,074	614	758	312	1.12636	809 (331)	864	17.9152
	*Erysipelatoclostridium ramosum* DSM 1402	ABFX00000000	Gram-positive	~3.2	3,041	535	658	221	1.17318			
	*Ruminococcus lactaris* CC59-002D	AZJE00000000	Gram-positive	~2.7	2,855	587	706	166	1.18649			
	*Clostridium clostridioforme* CM201	AGYS00000000	Gram-positive	~5.6	6,074	583	707	273	0.974817			
HC	*Coprococcus catus* GD/7	NC_021009	Gram-positive	~3.5	2,972	551	706	197	36.1197	899 (380)	930	7.41658
	*Ruminococcus bromii* AM32-13AC	QSIY01000000	Gram-positive	~2.5	2,007	459	598	134	27.6451			
	*Coprococcus eutactus* 2789STDY5834963	CYXU00000000	Gram-positive	~3.1	2,665	585	722	179	27.9975			
	*Gemmiger formicilis* ATCC 27749	FUYF00000000	Gram-negative	~3.2	2,882	524	650	178	0.616684			
UC	*Ruminococcus callidus* ATCC 27760	AWVF01000000	Gram-positive	~3.1	2,791	493	621	156	1.18834	871 (368)	915	3.23853
	*Faecalibacterium prausnitzii* A2165	NZ_CP022479	Gram-negative	~3.1	2,956	528	655	177	20.9628			
	*Clostridium celatum* DSM 1785	AMEZ01000000	Gram-positive	~3.5	3,211	580	698	228	22.7295			
	*Ruminococcus albus* 7 DSM_20455	NC_014833	Gram-positive	~4.4	3,983	555	680	197	1.15961			

* *The number given in the brackets are number of reactions with fluxes*.

# *FBA- Flux Balance Analysis*.

Taken together, our analysis supported the initial postulation that the gut dysbiosis presents itself in a disease-specific manner and can be harnessed for diagnostic and/or prognostic purposes. Therefore, we further investigated to determine if the metabolic profiles of the above-identified microbial species also confer specificity for CD, UC, and HC to help distinguish between the IBD disorders and with healthy controls.

### Validation of Disease-Specific Species Using Distinct Datasets

For the validation purpose, we have used the two whole metagenomics datasets (Supplementary Figure 4A). The alpha diversity (Shannon diversity) and beta diversity (Bray-Curtis distance) were analyzed, which showed similar results with our previous comparisons. HC group showed higher Shannon diversity over both the CD and UC groups (Supplementary Figure 4B). Beta diversity was smaller when there was more overlap of species between CD and UC groups (Supplementary Figure 4C). We analyzed the order- and species-level comparisons for CD vs. HC, UC vs. HC, and CD vs. UC (Supplementary Datasheet 8). In the prior comparison, members of order *Bacteroidales* and *Clostridiales* were enriched in all the three comparisons and a similar trend was observed in these datasets too (Supplementary Figures 5A–C). Similarly, at the species-level, in comparison to the previously identified significant OTUs, seven out of seven in CD vs. HC (Supplementary Figure 6A), 11 out of 12 in UC vs. HC (Supplementary Figure 6B) and ten out of ten in CD vs. UC (Supplementary Figure 6C) were also identified in these two datasets (Supplementary Datasheet 8). These results using distinct datasets validate our prior results using five datasets and demonstrate that the disease-specific species identified in this study can be reliably advanced to metabolic modeling studies.

### Metabolic Modeling Using the Pan-Genomic and Pan-Metabolomic Data

The 12 disease-specific microbial species that we identified in CD, UC, and HC cohorts showed a large variation in their genome size, indicating a diverse metabolic footprint across the organisms. *R. bromii* and *C. bolteae* contained the smallest and largest genomes (at ~2.5 and ~ 6.6 Mb), respectively (Table 1). First, we looked at the genome-level similarities among these 12 species using the Gegenees similarity analysis tool, which showed the similarity range between 18 and 78% at the nucleotide level (Supplementary Figure 2). Then, species-level metabolic models were reconstructed for all 12 organisms by choosing appropriate templates from the Gram-positive or Gram-negative species. These predicted models are provided in the SBML (.xml) and excel (.xls) formats in the Supplementary Folder: Model.zip.

For each of the 12 reconstructed metabolic models, we identified all possible biological reactions and chemicals/metabolites involved in the complete reaction. These reactions included forward, reverse as well as bi-directional biological reactions. The total number of genes, reactions, and metabolites that are potentially involved in these metabolic models, for all the 12 microbial genomes, are listed in Table 1. The combined set of genes, reactions and metabolites from each group were then used for CD vs. HC, UC vs. HC and CD vs. UC comparisons, to identify the pan, core, accessory and unique sets of genes, and corresponding reactions and metabolites (Supplementary Table 4). To identify the reactions that are specific to CD, UC, and HC cohorts, we excluded all the core reactions that are present in all 12 genomes and separated the unique and accessory reactions that are exclusive to each cohort. Likewise, we identified disease-specific or control-specific genes and metabolites. From these metabolic models, we obtained the number of specific reactions, metabolites and genes in each diseased condition (CD and UC) and healthy control (HC). However, only a limited number of the specific reactions were present within the communities of CD, UC, and HC when compared with each other (Supplementary Table 5). For example, in comparison of the CD vs. HC, only 141 reactions were identified as CD specific. Likewise, in UC vs. HC, 153 reactions were identified as UC specific. While comparing disease associated reactions, CD vs. UC 124 and 186 reactions were identified as specific to CD and UC, respectively. Since the identified disease-specific microbes belonged to a different genus, there are many reactions that were identified as single specific reactions in each metabolic model, even though they were not shared with their community. Similarly, we compared the metabolites and genes involved in the metabolic models and the total numbers of identified items have been listed in Supplementary Table 5. The entire list of the reactions, compound and genes in the metabolic model and their specific reactions, compound and genes, which differentiate CD vs. HC, UC vs. HC and CD vs. UC, are provided in Supplementary Datasheet 6.

### Community Metabolic Modeling Using Disease-Specific Microbes

**Figure 5:**
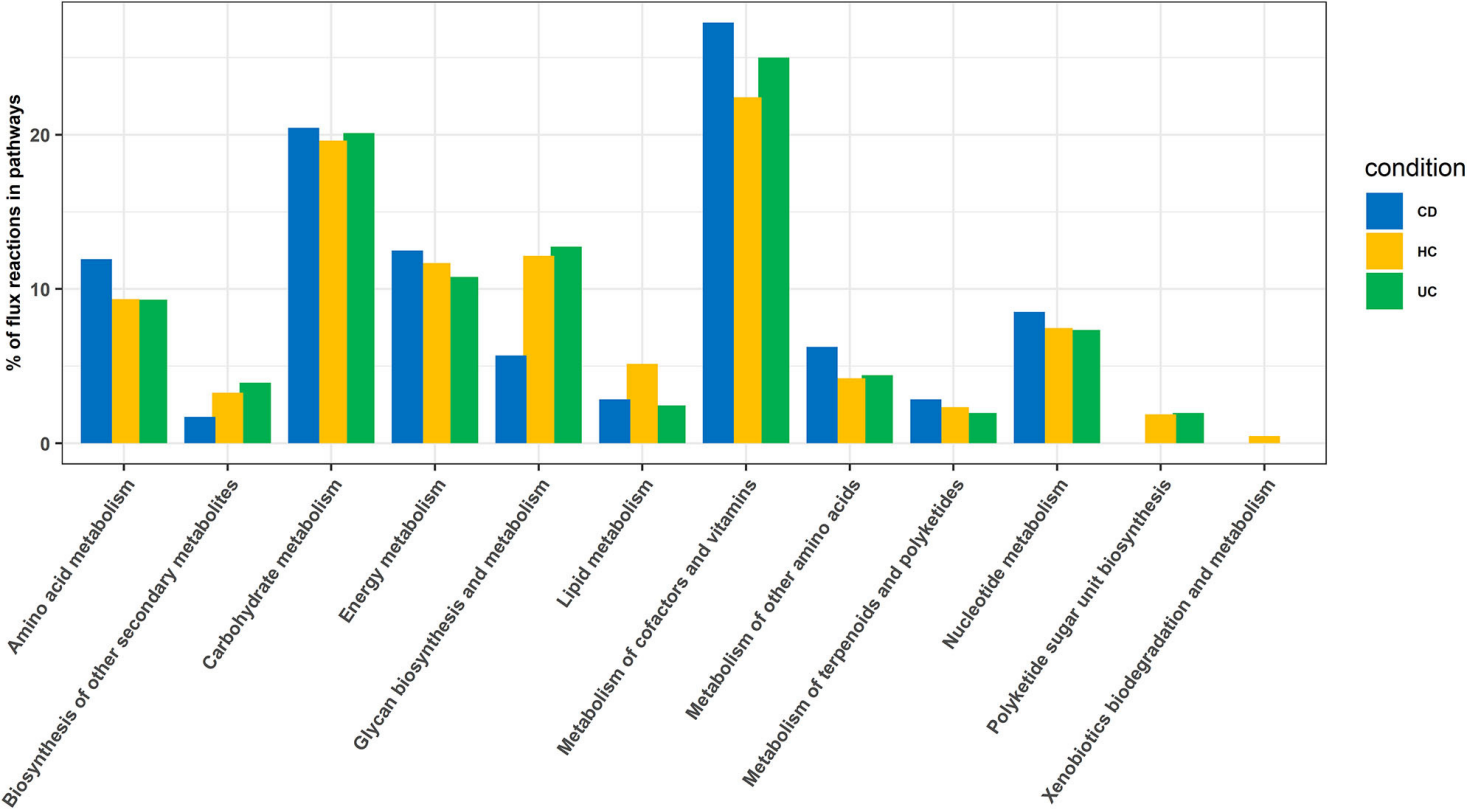
Enriched pathways identified based on enriched metabolic reactions in disease-specific organisms for HC, CD, and UC cohorts.

In this analysis, we combined the metabolic models of all organisms in each cohort to build a community model for each of the CD, UC and HC cohorts. For example, metabolic models of *C. bolteae, C. ramosum, R. lactaris*, and *C. clostridioforme* were combined to generate a single community metabolic model for CD. These models are provided in SBML (.xml) and excel (.xls) formats in the Supplementary Folder: Model.zip. Notably, from the CD, HC, and UC comparisons, the total identified reactions from the community model were 809, 899, and 871, respectively. To further determine the reaction fluxes, flux balance analysis was performed for each community model with a goal to determine the maximum reaction biomass for each model. The growth rate of the biomass yield for CD, HC, and UC showed the objective values as 17.91, 7.41, and 3.2, respectively (Table 1 and Supplementary Datasheet 7). Here, the identified metabolites in CD were highly enriched in pathways including metabolism of the cofactors and vitamins, amino acid metabolism, metabolism of other amino acids, and metabolism of terpenoids and polyketides. However, the UC metabolites were enriched more in the glycan biosynthesis and metabolism, biosynthesis of the other secondary metabolites, and polyketide sugar unit biosynthesis pathways (Figure 5). On the other hand, metabolic pathways such as lipid metabolism and xenobiotic biodegradation and metabolism were rather high in the HC, while pathways relating to the carbohydrate metabolism, nucleotide metabolism, and energy metabolism were equally distributed in all three groups. We also identified that there were 331, 380 and 368 enhanced flux reactions involved in 44, 55 and 47 sub-pathways of CD, HC, and UC, respectively (Supplementary Figure 3). Based on the flux values and their reactions, we then compared the HC, UC, and CD to detect cohort specific reactions (Supplementary Datasheet 6). Interestingly, these comparisons led to the identification of specific metabolic reactions that differentiate for CD, UC, and HC (Table 2).

**Table 2:** Differential microbiome patterns, metabolites, and metabolic function changes in CD vs. UC.

**Disease**	**Differential microbiota change**	**Key enzymes involved**	**Functions**
UC	*Ruminococcus albus Ruminococcus callidus Clostridium celatum Faecalibacterium prausnitzii*	Pyruvate synthase (S)-Malate:NADP+ oxidoreductase(oxaloacetate-decarboxylating) CoA-transferases glycoside hydrolases D-glucose 1-epimerase Cellulases Galactosidase	Digestion of plant fibers Cellulose metabolism Starch degradation Glycan degradation Decreases pro inflammatory cytokines Methane production Reduces nitrate to nitrite Hydrolyse Hippurate and starch Involved in glucose and mucin production Anti-inflammatory effect T-reg cells regulation
CD	*Clostridium bolteae Clostridium ramosum Ruminococcus lactaris Ruminococcus callidus*	sn-Glycero-3-phosphocholine glycerophosphohydrolase D-psicose 3-epimerase isocitrate lyase malate synthase D-glyceraldehyde-3-phosphate aldose-ketose-isomerase 4-Carboxymuconolactone carboxy-lyase L-Rhamnose ketol-isomerase NAD-dependent threonine 4-phosphate dehydrogenase Pyridoxamine-5′-phosphate:oxygen oxidoreductase D-Galactonate hydrolyase (R)-Glycerate:NAD+ oxidoreductase	Lower carbohydrate oxidation Increased fat oxidation (Reduced fat accumulation) Involved in tryptophan metabolism Involved in polyamines metabolism Increases production of enterolignans, enterodiol and enterolactone from plant lignin Involved in lactose (important enzyme: Galactosidase) and fructose metabolism Increased production of butyrate (acetyl Co A, glutrate, lysine and amino butyrate pathways) Negatively regulate leucine and bile acids Increased fat transporter Involved in wound healing, neutrophil recruitment and intestinal motility Stimulate the production of pro inflammatory cytokines

### The Disease-Specific Gut Microbiome Affects Specific Host Metabolic Pathways

We found disease-specific enrichment of the gut microbial communities in IBD compared to HC. Therefore, we further examined specific metabolic pathways that can be altered based on the microbial communities specific to UC and CD cohorts (Table 2). Also, to understand the potential impact on the host metabolism due to disease-specific enrichment of microbial communities, we explored the metabolic footprints of these communities. As expected, our meta-analysis showed that microbial species unique to HC are involved primarily in the breakdown of non-digestible carbohydrates and resistant starch alongside generation of lactate, acetate, propionate, and butyrate. However, the microbial communities differentially enriched in CD patients (vs. UC) potentially impact the higher carbohydrate utilization as reflected by the enrichment of pathways involved in the metabolism of simple carbons such as fructose, mannose, and galactose (Supplementary Figure 3 and Table 2). Also, glyoxylate and dicarboxylate metabolic pathways involved in carbohydrate biosynthesis from the fatty acids were increased in association with differential enrichment of the CD microbiota vs. UC (Supplementary Figure 3 and Table 2). Benzoate degradation, a metabolic process associated with the induction of inflammation, was also upregulated specifically in the CD. Interestingly, the microbiota enriched in the CD also exhibited increased antioxidant defense molecule processing, including ascorbate and glutathione metabolism (Supplementary Figure 3 and Table 2). On the other hand, UC enriched microbiota were associated with an increase in the metabolic pathways related to glycolytic and gluconeogenic metabolic pathways that are involved in maintaining the normal energy hemostasis. We also found that the pyruvate metabolic pathway was increased in the UC enriched microbiota compared to the CD enriched microbiota (Supplementary Figure 3 and Table 2). Overall, our data suggested that disease-specific enrichment of microbial communities affect the host metabolic pathways in disease-specific manners.

## Discussion

Our study represents one of the first efforts to discover the IBD-associated microbes and cohort-specific reactions from 16S rRNA and whole metagenome datasets using computational methods. Microbiota diversity has been known to play a key role in IBD (26). Earlier studies have shown an association between salmonella and campylobacter infections with an increased risk of IBD (27). However, another report did not show any consistent association between *Mycobacterium avium* subspecies *paratuberculosis* with CD (28). Some viruses, including the measles virus, were initially thought to be a risk factor for IBD (29). Later, *Clostridioides difficile*, cytomegalovirus infection, and other causes of sepsis have been noted to cause exacerbation of IBD, but no causal link has been detected (30). As mentioned before, UC and CD are sufficiently different in their pathobiology despite the similarities in disease symptoms and pathologies (31). Multiple studies have observed significant differences in the gut intestinal microbiomes of IBD patients when compared to the healthy individuals (2, 32, 33). These studies have led to the general perception that dysregulation of gut microbial diversity is potentially similar in CD and UC patients, and is characterized by a lower proportion of the *Firmicutes* and an increase in Gamma proteobacteria (34).

Due to the high prevalence of IBD in the developed countries, we performed data analysis on IBD samples (with at least 20 patient samples in each of the CD, UC, and HC cohorts) only from the developed countries. First, we looked at the alpha and beta diversity of the samples and cohorts using the Shannon index and Bray-Curtis distance measure, respectively. As expected, the alpha diversity trended higher in most of the health control datasets compared to the two IBD groups (CD and UC) (Figure 1A). Likewise, beta diversity as measured by the Bray-Curtis distance measure between the cohorts showed notable differences (Figure 1B) with the highest beta diversity recorded in CD vs. UC comparison and the lowest in HC vs. UC. These results indicate that there is only a small overlap of microbial species between CD and UC, which supports our notion that gut dysbiosis precipitates in a disease-specific manner. On the other hand, there's relatively a higher overlap of microbial species (less beta diversity) between UC and HC samples indicating that the UC microbiome is relatively closer to healthy controls compared to that of CD.

Then, we looked at the detailed profiles of bacterial species at different hierarchical taxonomic levels (kingdom to species) between the disease and healthy cohorts. Because the differences are minimal at the higher taxonomic levels, we focused on the profiles at the order level and below. Specific differences in microbes were noted by comparing the healthy and disease cohorts in three different ways, i.e., HC vs. IBD (Figure 2); HC vs. CD vs. UC (Figure 3); and CD vs. UC (Figure 4). Using a strict criteria that a species must be present in at least three out of the five datasets analyzed, we identified a combined 12 different species, four for each cohort that can be used as unique microbial markers (Supplementary Table 3). The genus *Clostridium* and *Ruminococcus* were highly prevalent in CD and UC, respectively. In HC, *Coprococcus* and *Gemmiger* played a vital role in differentiating healthy individuals from disease cohorts. Taken together, our results validated a similar outcome from other studies that the diversity of microbial communities is altered in IBD patients (9, 11). Similarly, He et al. compared 74 mucosal biopsies from 15 participants, including nine CD patients and six healthy individuals. They reported that 65 genera were identified as differentially abundant between active and quiescent CD, with a loss of *Fusobacterium* and a gain of potentially beneficial bacteria, *Lactobacillus, Akkermansia, Roseburia, Ruminococcus*, and *Lachnospira* after the induction of remission (35). These taxa also showed a positive correlation with clinical disease severity and a negative correlation with species richness. Our analysis also reported the *Clostridium* from two different families Lachnospiraceae and Clostridiaceae. It is noteworthy to point out that the UC-specific *C. celatum* is a member of the family Clostridiaceae while the two CD-specific Clostridium species are members of the family Lachnospiraceae (36). Similarly, *Ruminococcus* was also reported in two different families, Ruminococcaceae in UC and Lachnospiraceae in CD.

Our study noted that there are significant changes in *F. prausnitzii*, which differentiate the UC patients from CD patients. Of interest, *F. prausnitzii*, the most abundant bacterium in the healthy human gut is the major member of the Firmicutes phylum (37). Importantly, *F. prausnitzii* has immune-suppressive effects. It produces a protein that inhibits the NF-κB pathway, stimulates production of anti-inflammatory cytokine IL-10, and inhibits ulcerative colitis in BALB/c mice (37). *F. prausnitzii* is depleted in several intestinal disorders; however, more consistently in CD patients (38). Our analysis confirmed similar depletion of this microbial species in the CD patients. However, it revealed a contrasting enrichment in the UC patients. Notably, *F. prausnitzii* also produces the short-chain fatty acid, butyrate, an essential nutrient for the intestinal epithelial cells and its increase in UC patients may represent an adaptive enrichment. Furthermore, the proportions of the Clostridia were altered in CD patients: the Roseburia and *Faecalibacterium* genera of the *Lachnospiracae* and *Ruminococcaceae* families were decreased while *R. gnavus* was increased (32).

Comparison of the genome size and sequence similarities among the twelve species (Supplementary Figure 2 and Table 1) revealed vast variations. The sequence similarity between some species was as low as 40% indicating that the diversity of these genomes also contributes to a diverse metabolic footprint that affects the host metabolism in a disease-specific manner. Remarkably, several recent studies suggest that microbial diversity affects disease conditions by impacting the host-microbe interaction in regulating the host metabolism (39). To understand these interactions, we further analyzed the metabolic profiles of disease-specific species that we identified above using metabolic modeling and flux balance analysis. We identified significant pathways in CD and UC, which included enriched pathways related with amino acid and Glycan biosynthesis and metabolism.

Studies have shown that gut microbiota impact the host potentially by influencing the metabolism by producing specific enzymes and/or metabolites (40, 41). Interestingly in our findings, species unique to the HC are involved primarily in the breakdown of non-digestible carbohydrates and resistant starch, and the generation of short-chain fatty acids. Of interest, butyrate plays a crucial physiological role in maintaining the health and integrity of the colonic mucosa (42). CD enriched microbial species were mostly involved in fructose, mannose, and galactose metabolism. In this regard, *C. bolteae* and *R. callidus* enriched in CD are known to use above sugars and metabolize them into glyceraldehyde-3 phosphate, a key metabolite of the glycolytic pathway, the principal energy-generating mechanism in human body (43). Additionally, the glutathione and ascorbate pathways, involved in the maintenance of normal homeostasis during oxidative stress, were enriched in CD.

In comparison, the UC enriched microbiota are associated with an increase in the glycolytic, gluconeogenic, and pyruvate metabolic pathways. Notably, pyruvate can be catabolized into succinate, lactate, or acetyl-CoA and can be metabolized into acetate, propionate, and butyrate (43). We speculate these changes will help promote adaptive responses against inflammatory insults to heal the mucosa. *F. prausnitzii*, a “health-promoting” microbiota, was also explicitly increased in the UC patients. Studies have reported anti-inflammatory properties of this microbiota by promoting IL-10 production while and inhibiting NF-kB activity in the host cells. Also, *F. prausnitzii* is linked with butyrate production (37). Taken together, our data suggested that the enzymes involved in specific host metabolic pathways can be impacted differentially by the gut microbiota in CD vs. UC, though a systematic experimental investigation is warranted to uncover further details. This study supports the identification of disease-specific microbial communities and their effects on the host metabolism, which helps researchers differentiate between IBD (CD and UC) diseases in the initial stages.

## Conclusions

In conclusion, this article represents an unbiased determination of the relative status of the gut microbial communities in IBD patients compared with healthy controls, using meta-analysis of five different IBD datasets available in the public domain representing populations from five different developed countries. While this analysis confirmed the generally recognized association of the gut microbial dysbiosis with IBD, it also revealed that this dysbiosis bears disease specificity, as we found significant changes in microbiota enrichment in UC vs. CD at different taxonomic levels down to the genus and species. The metabolic modeling further demonstrated the significance of dynamic host-microbe interactions in affecting host metabolism, which potentially is mediated by the release of specific microbial enzymes and metabolites. We believe that such information will not only help development of potential biomarkers for disease validity in non-invasive manner but also therapy response. Obviously, further detailed analysis is needed to satisfy such needs and is part of our ongoing studies.

## Data Availability Statement

The original contributions presented in the study are included in the article/Supplementary Materials, further inquiries can be directed to the corresponding author/s.

## Author Contributions

AS and CG supervised the study. JS, AS, and CG designed experiments. JS, RA, and NA generated and analyzed data. JS, NA, and CG made the figure panels. JS and RA wrote the original draft. NA, AS, and CG reviewed and edited the manuscript. All authors have read and approved the final manuscript.

## Conflict of Interest

The authors declare that the research was conducted in the absence of any commercial or financial relationships that could be construed as a potential conflict of interest.

## Supplementary Material

Supplementary Folder Model.zip. This folder contains the predicted model for 12 organisms and the community model of CD, HC, and UC in SBML (.xml) and Excel (.xls) format. Available online at: https://doi.org/10.6084/m9.figshare.13208204.v1.
