# Supplementary material for: Gut Microbiota and Metabolic Specificity in Ulcerative Colitis and Crohn's Disease
Source: Front Med (Lausanne). 2020 Nov 27;7:606298. doi: 10.3389/fmed.2020.606298 (PMC7729129; doi:10.3389/fmed.2020.606298)
Supplement: Supplementary Folder — Model.zip. This folder contains the predicted model for 12 organisms and the community model of CD, HC, and UC in SBML (.xml) and Excel (.xls) format. Available online at: https://doi.org/10.6084/m9.figshare.13208204.v1. [file Table_1.DOCX]

**Supplementary Table S1.** Significant OTUs at different taxonomic levels from kingdom to species**.**

| **OTUs classification** | **IBD vs. HC** | **CD vs. UC** | **CD vs. UC vs. HC** |
| --- | --- | --- | --- |
| Kingdom | 3 | 1 | 2 |
| Phylum | 9 | 8 | 11 |
| Class | 16 | 14 | 18 |
| Order | 25 | 30 | 28 |
| Family | 37 | 32 | 40 |
| Genus | 55 | 53 | 61 |
| Species | 66 | 47 | 75 |

**Supplementary Table S2**. Identification of significantly different order-level OTUs between different comparison groups.

| **Datasets / classification** | **Significance** | | |
| --- | --- | --- | --- |
|  | **IBD vs. HC** | **CD vs. UC** | **CD vs. UC vs. HC** |
| SRP183770 | 28 | 29 | 10 |
| SRP128892 | 63 | 77 | 109 |
| ERP008725 | 11 | 33 | 40 |
| SRP115494 | 18 | 21 | 27 |
| SRP129027 | 58 | 88 | 41 |
| Conserved | 7 | 10 | 12 |
| Total | 146 | 195 | 168 |

**Supplementary Table S3.** List of disease-specific organisms used for building metabolic reconstruction models.

| **Diseases** | **Kingdom** | **Phylum** | **Class** | **Order** | **Family** | **Organism (Genus species)** |
| --- | --- | --- | --- | --- | --- | --- |
| CD | Bacteria | Firmicutes | Erysipelotrichi | Erysipelotrichales | Erysipelotrichaceae | *Clostridium ramosum* |
|  | Bacteria | Firmicutes | Clostridia | Clostridiales | Lachnospiraceae | *Ruminococcus lactaris* |
|  | Bacteria | Firmicutes | Clostridia | Clostridiales | Lachnospiraceae | *Clostridium clostridioforme* |
|  | Bacteria | Firmicutes | Clostridia | Clostridiales | Lachnospiraceae | *Clostridium bolteae* |
| HC | Bacteria | Firmicutes | Clostridia | Clostridiales | Lachnospiraceae | *Coprococcus catus* |
|  | Bacteria | Firmicutes | Clostridia | Clostridiales | Ruminococcaceae | *Ruminococcus bromii* |
|  | Bacteria | Firmicutes | Clostridia | Clostridiales | Lachnospiraceae | *Coprococcus eutactus* |
|  | Bacteria | Firmicutes | Clostridia | Clostridiales | Ruminococcaceae | *Gemmiger formicilis* |
| UC | Bacteria | Firmicutes | Clostridia | Clostridiales | Ruminococcaceae | *Ruminococcus albus* |
|  | Bacteria | Firmicutes | Clostridia | Clostridiales | Ruminococcaceae | *Ruminococcus callidus* |
|  | Bacteria | Firmicutes | Clostridia | Clostridiales | Ruminococcaceae | *Faecalibacterium prausnitzii* |
|  | Bacteria | Firmicutes | Clostridia | Clostridiales | Clostridiaceae | *Clostridium celatum* |

**Supplementary Table S4**. Pan genome and metabolomics analysis for species-specific genes

|  | **CD *versus*** **HC** | **UC *versus* HC** | **CD *versus* UC** |
| --- | --- | --- | --- |
| **Genome** | | | |
| Pan | 17,863 | 16,878 | 20,256 |
| Core | 149 | 118 | 123 |
| Accessory | 3,959 | 1,821 | 3,955 |
| Unique | 13,755 | 14,939 | 16,178 |
| **Reaction involved in metabolic model** | | | |
| Pan | 1,040 | 1,052 | 995 |
| Core | 243 | 229 | 275 |
| Accessory | 584 | 565 | 516 |
| Unique | 213 | 258 | 204 |
| **metabolites involved in metabolic model** | | | |
| Pan | 896 | 902 | 883 |
| Core | 398 | 383 | 413 |
| Accessory | 406 | 391 | 366 |
| Unique | 92 | 128 | 104 |
| **Genes involved in metabolic model** | | | |
| Pan | 841 | 802 | 886 |
| Core | 14 | 13 | 23 |
| Accessory | 256 | 200 | 269 |
| Unique | 571 | 589 | 594 |

**Supplementary Table S5**. Identification of specific reactions, metabolites and genes from metabolic models.

| **Disease/**  **Control** | **CD *versus*** **HC** | | **UC *versus* HC** | | **CD *versus* UC** | |
| --- | --- | --- | --- | --- | --- | --- |
|  | **Identification of disease-specific reactions from the accessory reaction** | | | | | |
|  | CD specific | HC specific | UC specific | HC specific | CD specific | UC specific |
| CD | 141 | absent | 153 | absent | 124 | absent |
| HC | absent | 231 | absent | 181 | absent | 186 |
|  | **Identification of disease-specific metabolites from the accessory metabolites** | | | | | |
| UC | 73 | absent | 79 | absent | 68 | absent |
| HC | absent | 121 | absent | 87 | absent | 108 |
|  | **Identification of disease-specific accessory genes in the metabolic model** | | | | | |
| CD | 366 | absent | 306 | absent | 4033 | absent |
| UC | absent | 329 | absent | 319 | absent | 364 |
