# Supplementary material for: Gut Microbiota and Metabolic Specificity in Ulcerative Colitis and Crohn's Disease
Source: Front Med (Lausanne). 2020 Nov 27;7:606298. doi: 10.3389/fmed.2020.606298 (PMC7729129; doi:10.3389/fmed.2020.606298)
Supplement: Supplementary file 2 [file Data_Sheet_1.PDF]

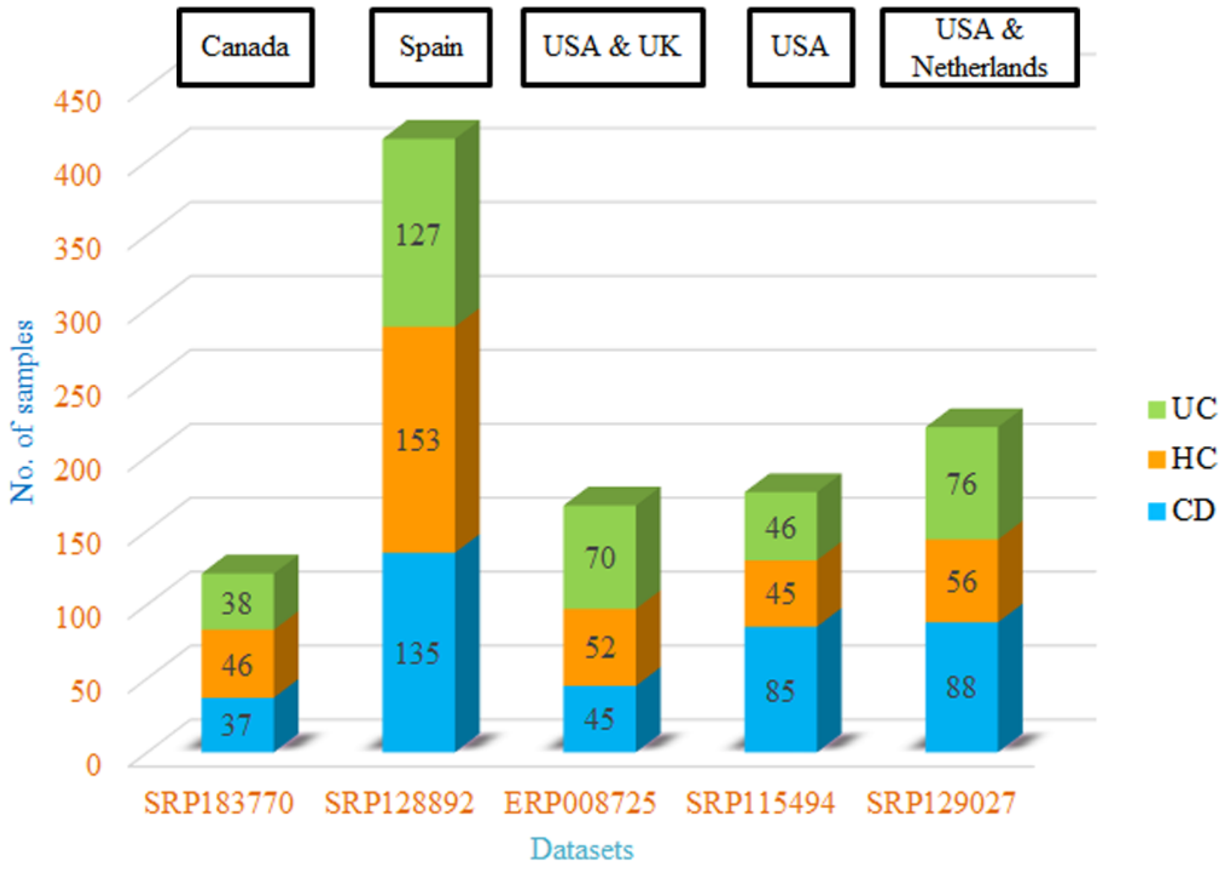

Supplementary Figure S1. Details of 16S rRNA and whole metagenome datasets used for the meta-analysis.

	Organism	CD				HC				UC			
		1	2	3	4	5	6	7	8	9	10	11	12
CD	1: Clostridium_bolteae_ATCC_BAA_613	100	66	58	78	45	53	46	56	41	67	52	41
	2: Erysipelatoclostridium_ramosum_DSM_1402	66	100	44	46	45	44	44	21	47	45	43	49
	3: Ruminococcus_lactaris_CC59_002D	63	49	100	65	74	68	65	62	51	65	53	50
	4: Clostridium_clostridioforme_CM201	78	50	67	100	51	65	54	68	68	63	55	48
HC	5: Coprococcus_catus_GD_7	44	40	73	44	100	57	74	66	45	78	44	45
	6: Ruminococcus_bromii_AM32_13AC	55	40	72	74	57	100	64	76	63	70	45	43
	7: Coprococcus_eutactus_2789STDY5834963	47	39	64	45	74	63	100	44	59	68	43	41
	8: Gemmiger_formicili_ ATCC_27749	53	22	57	72	64	73	48	100	74	58	18	28
UC	9: Ruminococcus_callidus_ATCC_27760	40	47	40	71	45	63	58	75	100	60	46	44
	10: Faecalibacterium_prausnitzii_A2165	64	48	64	64	77	67	67	61	66	100	49	55
	11: Clostridium_celatum_DSM_1785	51	37	42	48	45	46	40	18	49	48	100	51
	12: Ruminococcus_albus_7_DSM_20455	48	49	48	46	51	52	47	28	52	51	50	100

Supplementary Figure S2. Heatmap showing genomic similarity among twelve disease-specific organisms. The CD-, HC-, and UC-specific organisms are shown together in blue, orange and green boxes.

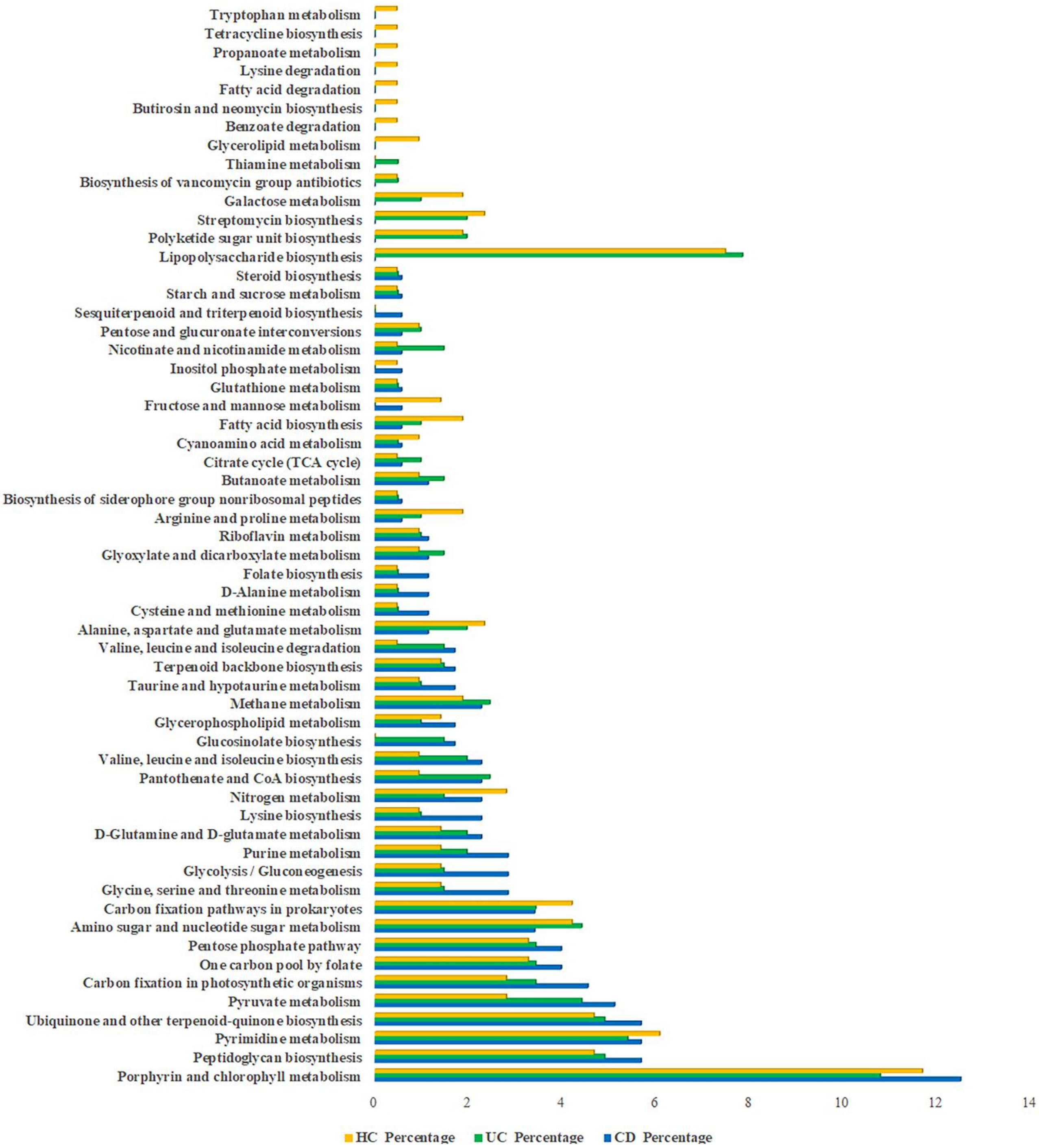

Supplementary Figure S3. In community model, the reactions which having flux's were identified and their pathways were identified and compared. A total of 44, 55 and 47 sub-pathways were identified in CD, HC and UC, respectively. These pathways showed a clear differentiation among CD, HC and UC.

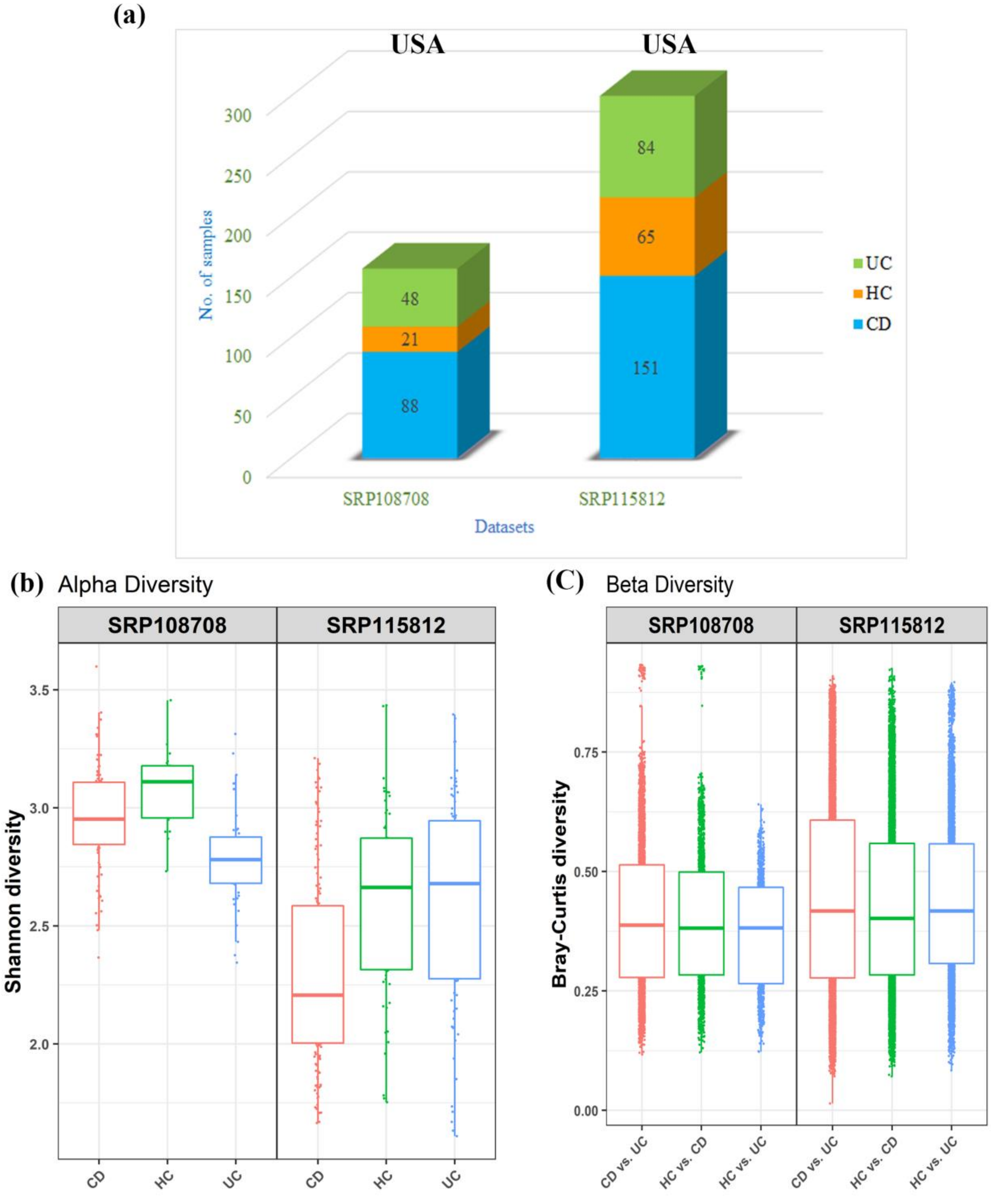

Supplementary Figure S4. (a) Details of whole metagenome datasets used for the meta-analysis validation. (b) Boxplot showing Shannon diversity of each group. Each dot represents a sample and the lines in the boxes correspond to the median of samples; (c) Bray–Curtis distances between the comparison pair. Dots represent the distance between the samples in each comparison group and the lines in the boxes correspond to the median.

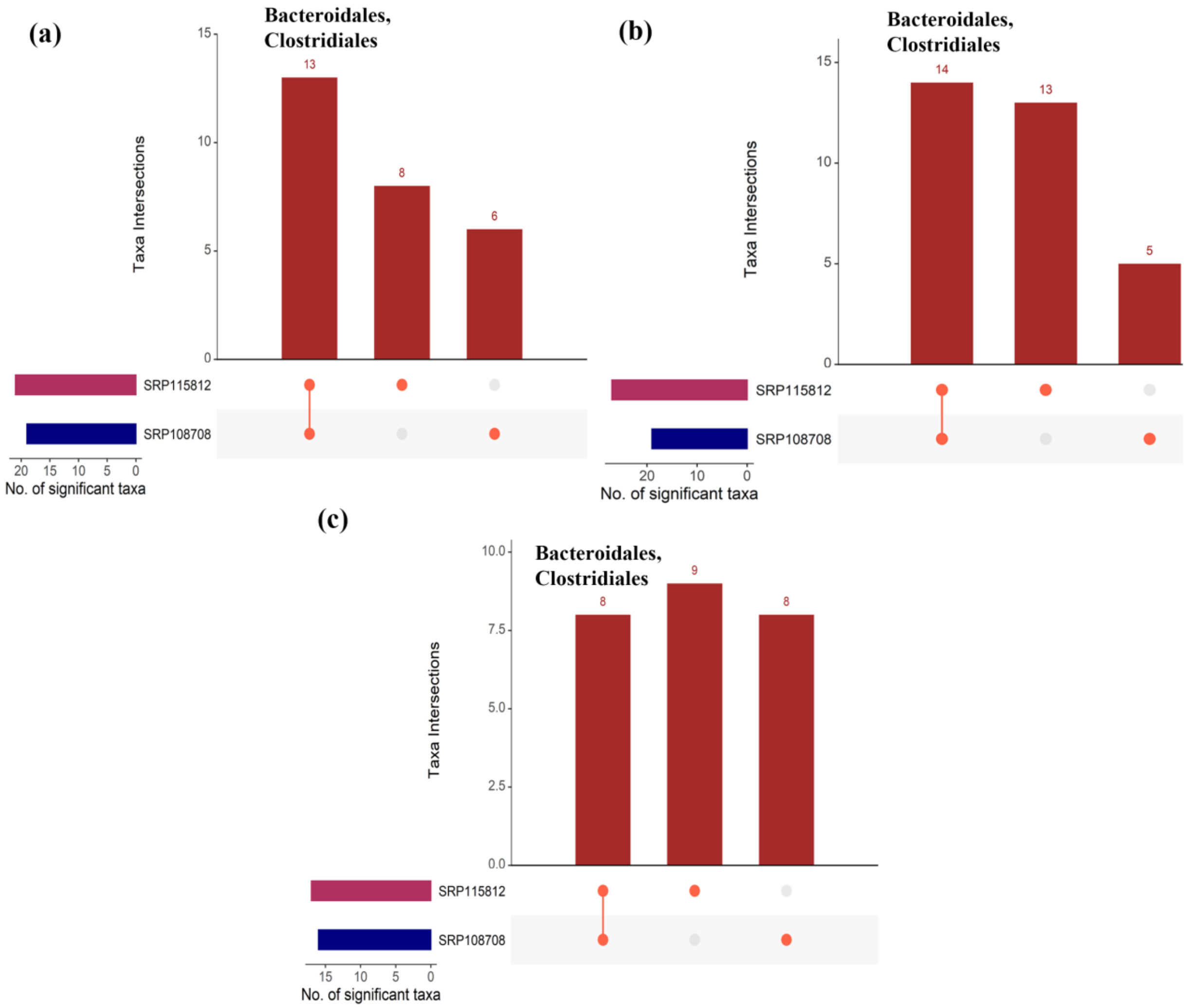

Supplementary Figure S5. An upset plot showing taxonomic intersections across the two datasets at the Order-level. (a) HC *versus* IBD (b) CD *versus* HC *versus* UC and (c) CD *versus* UC. Each bar represents the number of orders in that category and the orange dot below the bar indicates their conservation across the datasets. Here also instance, members of Clostridiales are conserved in all two datasets.

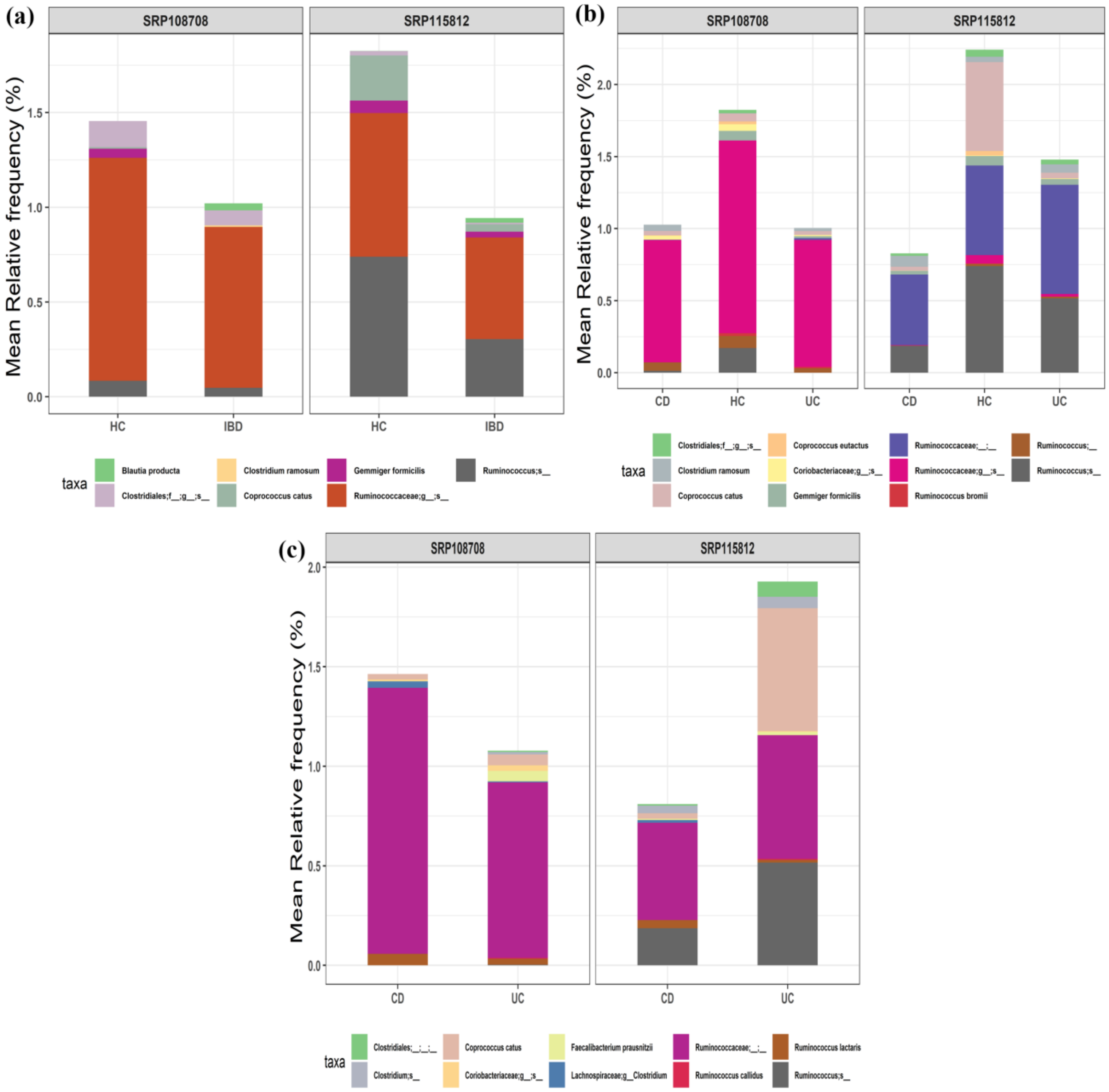

Supplementary Figure S6. Comparison of microbial communities across two datasets. Stacked bar plots show the relative mean frequencies of significant species-level communities Here we plotted only the previously identified OTUs in all the comparisons those OTUs were highlighted in 336_OTUs, 308_OTUs and 242_OTUs sheet in Supplementary File S7 (a) HC or IBD (b) CD, HC or UC (c) CD or UC.
